# Efficacy of the polyglycolic acid sheet for preventing anastomotic leakage in double-stapling technique anastomosis for left-sided colon or rectal cancer surgery: a propensity score-matched study

**DOI:** 10.1186/s12893-023-02044-0

**Published:** 2023-05-17

**Authors:** Masatsune Shibutani, Tatsunari Fukuoka, Yasuhito Iseki, Hiroaki Kasashima, Kiyoshi Maeda

**Affiliations:** Department of Gastroenterological Surgery, Osaka metropolitan university graduate school of medicine, 1–4–3 Asahi-machi Abeno-ku, Osaka City, 545-8585 Osaka Prefecture Japan

**Keywords:** Double-stapling technique, Anastomotic leakage, Polyglycolic acid sheet

## Abstract

**Background:**

To prevent anastomotic leakage in patients with left-sided colorectal cancer who underwent double-stapling technique (DST) anastomosis, we investigated a new method: DST anastomosis with a polyglycolic acid (PGA) sheet. This procedure has been shown to have the potential to decrease the rate of anastomotic leakage. However, due to the small number of cases enrolled in our previous study, it was not possible to compare the outcomes of the new and conventional procedures. The aim of this study was to evaluate the effect of the PGA sheet on preventing anastomotic leakage in patients with left-sided colorectal cancer who underwent DST anastomosis by retrospectively comparing the anastomotic leakage rate between the PGA sheet and conventional groups.

**Methods:**

A total of 356 patients with left-sided colorectal cancer who underwent DST anastomosis during surgery at Osaka City University Hospital between January 2016 and April 2022 were enrolled in this study. Propensity score matching was performed to reduce the confounding effects secondary to imbalances in the use of PGA sheets.

**Results:**

The PGA sheet was used in 43 cases (PGA sheet group) and it was not used in 313 cases (conventional group). After propensity score matching, the incidence of anastomotic leakage in the PGA sheet group was significantly lower than that in the conventional group.

**Conclusion:**

DST anastomosis with PGA sheet, which is easy to perform, contributes to the reduction of anastomotic leakage rate by increasing the strength of the anastomotic site.

## Background

Double-stapling technique (DST) anastomosis is a procedure that is frequently performed during surgery for left-sided colon or rectal cancer [1, 2]. However, the anastomotic leakage rate of DST anastomosis in rectal surgery is about 10% [3–5]. In addition to worsening the short-term outcomes, such as the re-operation rate and duration of hospitalization, anastomotic leakage has a negative impact on the oncological outcomes, such as recurrence rate and cancer-specific survival [6–8]. Therefore, various improvements in procedures and devices, including sufficient mobilization of the left-sided colon for tension-free anastomosis [9, 10], evaluation of intestinal perfusion by indocyanine green (ICG) fluorescence imaging [11–13], placement of a transanal tube to reduce intraluminal pressure [14–16], improvements in stapling devices [17–19], and intracorporeal reinforcement sutures [20, 21] have been made to prevent anastomotic leakage. However, anastomotic leakage remains a complication that needs to be resolved and continues to be a concern for colorectal surgeons. To solve this problem, we investigated a new method, DST anastomosis with a polyglycolic acid (PGA) sheet (Neoveil®; Gunze, Kyoto, Japan) [22]. This procedure has been shown to have the potential to decrease the rate of anastomotic leakage. However, due to the small number of cases enrolled in our previous study, it was not possible to compare the outcomes between the new and the conventional procedures.

The aim of this study was to evaluate the effect of PGA sheets on preventing anastomotic leakage in patients with left-sided colon and rectal cancer who underwent DST anastomosis by comparing the anastomotic leakage and re-operation rates between the PGA sheet and the conventional groups.

## Methods

### Patients

A total of 356 patients with left-sided colon or rectal cancer who underwent DST anastomosis during surgery at Osaka City University Hospital between January 2016 and April 2022 were enrolled in this study. Indocyanine green fluorescence imaging was performed during all operations; however, the left colic artery was not preserved in any of them. An expert in colorectal surgery participated in all surgeries. Accordingly, there was no operator bias in the use of the PGA sheet. For patients with intestinal obstruction, decompression was performed first by means such as a metallic stent, transanal tube, or colostomy, and then the primary lesion was resected. The following variables were analyzed: age, sex, body mass index, the American Society of Anesthesiologist physical status (ASA-PS), diabetes mellitus comorbidity, tumor depth, tumor diameter, tumor location, surgical approach (open/laparoscopic/robot-assisted), diverting ileostomy, number of stapler cartridges for rectal transection, duration of operation, intraoperative blood loss, neoadjuvant treatment, anastomotic leakage, re-operation, postoperative bleeding at the anastomotic site, and mortality.

### Surgical technique of the DST anastomosis with the PGA sheet

**Fig. 1:**
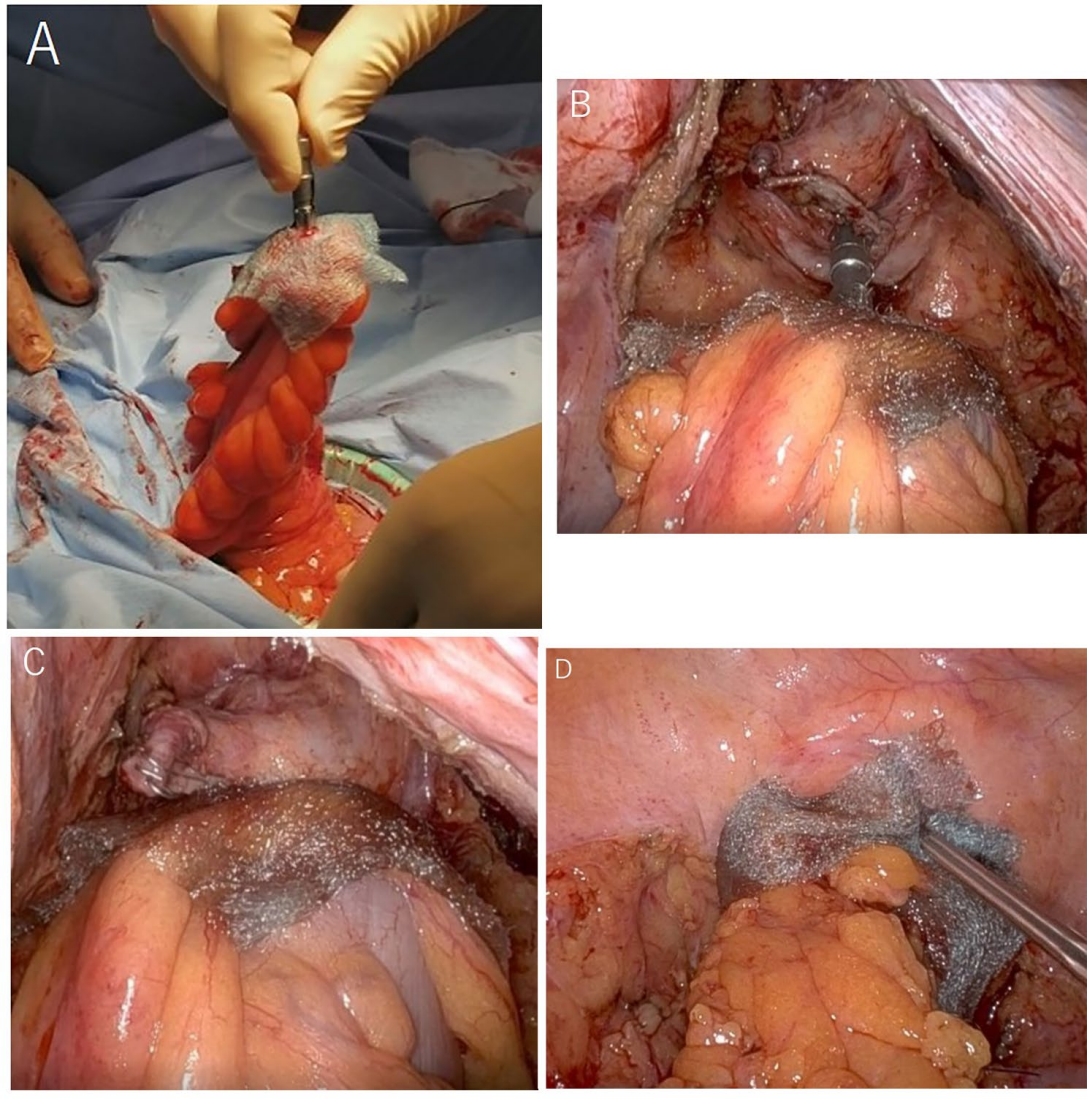
The outline of the double-stapling technique (DST) anastomosis with a polyglycolic acid (PGA) sheet (**A**) The PGA sheet was attached to the anvil. (**B, C**) DST anastomosis was performed with the PGA sheet sandwiched. (**D**) A strip of the PGA sheet was wrapped around the anastomosis

Details regarding the method of DST anastomosis with PGA sheets have been described in our previous report [22]. The remainder of this paper is organized as follows: First, a PGA sheet with a slit of a few millimeters was attached to the anvil (Fig. 1A). DST anastomosis was then performed with the PGA sheet sandwiched (Fig. 1B C). Finally, a strip of PGA sheet was wrapped around the anastomosis (Fig. 1D).

No changes were made during the study period in postoperative management, such as postoperative resumption of diet or placement of a transanal drainage tube.

### Definition of anastomotic leakage

Anastomotic leakage was defined as an extravasation observed on radiography. Upon observing the clinical signs of leakage, such as abdominal pain, high fever, leukocytosis, or pus/fecal discharge from the pelvic drain, computed tomography (CT) was performed to confirm the presence of anastomotic leakage. The following CT findings were considered suggestive of anastomotic leakage: abscess, fluid collection, or air bubbles surrounding the anastomotic site.

### Ethics statement

This retrospective study was approved by the Ethics Committee of Osaka City University (approval number: 4182) and was conducted in accordance with the Declaration of Helsinki. Written informed consent was obtained from all patients.

### Statistical analyses

All statistical analyses were performed using the SPSS software package for Windows (IBM, Chicago, IL, USA). The significance of differences between the use of PGA sheet and the patients’ background/operative outcomes were analyzed using a chi-squared test, Fisher’s exact test, and the Mann–Whitney U test. *P* < 0.05 was considered to be statistically significant. To reduce the impact of selection bias and potential confounding, which is associated with non-randomized observational studies, we performed propensity score matching. The propensity scores were estimated using multivariate logistic regression models, with the groups as the dependent variable and patient characteristics and operative outcomes as covariates. Matching was performed with a one-to-one greedy nearest neighbor algorithm with a caliper of 0.2 without replacement.

## Results

PGA sheets were used in 43 cases (PGA sheet group) while conventional techniques were used for the remaining 313 cases (conventional group). Patient characteristics are summarized in Table 1 and the operative outcomes are summarized in Table 2. Differences in patient characteristics and operative outcomes between the two groups before propensity score matching were observed for body mass index and duration of operation. Propensity score matching was performed using these factors as covariates.

**Table 1:** Patients’ characteristics and clinicopathological factors

	Entire cohort			Matched cohort		
Factors	The conventional group (n = 313)	The PGA sheet group (n = 43)	p-Value	The conventional group (n = 43)	The PGA sheet group (n = 43)	p-Value
Age (years)						
Median (range)	69 (31–92)	70 (42–88)	0.904	69 (31–90)	70 (42–88)	0.691
Sex, n						
Male	192	24		17	24	
Female	191	19	0.508	26	19	0.827
Body mass index (kg/m^2^)						
Median (range)	22.63 (14.55–40.88)	20.89 (14.84–32.55)	0.016	21.44 (14.55–31.31)	20.89 (14.84–32.55)	0.839
ASA physical status, n						
1, 2	267	33		37	33	
3	44	10		6	10	
4	2	0	0.161	0	0	0.407
Diabetes mellitus, n						
Negative	249	38		38	38	
Positive	64	5	0.218	5	6	> 0.999
Tumor location, n						
Descending colon	1	1		0	1	
Sigmoid colon	147	25		21	25	
Rs	68	6		7	6	
Ra	54	8		10	8	
Rb	43	3	0.187	5	3	0.709
Tumor diameter (cm)						
Median (range)	3.5 (0–11.0)	3.8 (0–12.0)	0.629	3.5 (0-7.5)	3.8 (0–12.0)	0.704
Tumor depth, n						
Tis	5	1		0	1	
T1	50	4		7	4	
T2	5	5		7	5	
T3	151	26		20	26	
T4	39	4		5	4	
After endoscopic resection	22	3		4	3	
Complete response after NACRT	1	0	0.807	0	0	0.671
Neoadjuvant treatment, n						
None	301	43		42	43	
Chemoradiotherapy	12	0	0.374	1	0	> 0.999

PGA, polyglycolic acid; ASA, American Society of Anesthesiologists; NACRT, neoadjuvant chemoradiotherapy; Ra, tumor above the peritoneal reflection; Rb, tumor at or below the peritoneal reflection

**Table 2:** Operative outcomes

	Entire cohort			Matched cohort		
Factors	The conventional group (n = 313)	The PGA sheet group (n = 43)	p-Value	The conventional group (n = 43)	The PGA sheet group (n = 43)	p-Value
Surgical approach, n						
Open	28	3		4	3	
Laparoscopic	255	36		34	36	
Robot-assisted	30	4	0.907	5	4	0.856
Number of stapler cartridges for rectal transection, n						
1–2	305	42		42	42	
> 2	8	1	> 0.999	1	1	> 0.999
Operative duration (min)						
Median (range)	217 (146–691)	189 (117–1129)	0.001	224 (136–574)	189 (117–1129)	0.076
Intraoperative blood loss (ml)						
Median (range)	20 (5-3700)	20 (5-2730)	0.299	10 (5-3700)	20 (5-2730)	0.826
Diverting ileostomy, n						
Absent	271	41		37	41	
Present	42	2	0.137	6	2	0.265

PGA, polyglycolic acid

Postoperative complications are summarized in Table 3. Before propensity score matching, the incidence of anastomotic leakage in the PGA sheet group was significantly lower than that in the conventional group (2.3% versus 13.4%, p = 0.042), and even after propensity score matching, similar result was observed. Before propensity score matching, re-operation was performed in 2.6% of the patients in the conventional group, whereas no re-operation was performed in the PGA sheet group, however, no statistically significant difference was observed. Furthermore, before propensity score matching, anastomotic bleeding was observed in 1.6% of the patients in the conventional group, whereas no anastomotic bleeding was observed in the PGA sheet group.

**Table 3:** Postoperative complication

	Entire cohort			Matched cohort		
Factors	The conventional group (n = 313)	The PGA sheet group (n = 43)	p-Value	The conventional group (n = 43)	The PGA sheet group (n = 43)	p-Value
Anastomotic leakage, n(%)	42 (13.4%)	1 (2.3%)	0.042	8 (18.6%)	1 (2.3%)	0.030
Re-operation, n(%)	8 (2.6%)	0 (0%)	0.603	1 (2.3%)	0 (0%)	> 0.999
Postoperative bleeding at anastomotic site, n(%)	5 (1.6%)	0 (0%)	< 0.999	0 (0%)	0 (0%)	N/A
Mortality, n(%)	0 (0%)	0 (0%)	N/A	0 (0%)	0 (0%)	N/A

PGA, polyglycolic acid; N/A, not applicable

## Discussion

This study demonstrated that PGA sheets contribute to a decrease in the risk of anastomotic leakage in patients with left-sided colon or rectal cancer who underwent DST anastomosis. Since DST anastomosis with a PGA sheet is easy to perform and the material is relatively inexpensive, this procedure has the potential to make anastomotic leakage a rare complication.

The PGA sheet, which is an absorbable reinforcement material, increases physical pressure resistance by forming a barrier due to thickening of the collagen tissue during hydrolysis [23, 24]. This material has been widely used in daily practice to prevent air leakage after lung surgery [25, 26] and to prevent pancreatic fistula after pancreatic surgery [27, 28]. As there have been no experimental animal studies, the effect of PGA sheets on fibrosis in the gastrointestinal tract is unknown. Nonetheless, it is speculated that the PGA sheet can exert its protective effect in the gastrointestinal tract, given that PGA sheets are effective in lung surgery, which deals with air that requires stricter control than liquids. Furthermore, in the previous basic research, PGA sheets have been reported to contribute to an increase in physical pressure resistance by stabilizing staples [29, 30]. As described above, PGA sheets may be useful in preventing anastomotic leakage; however, very few clinical studies have been conducted on DST anastomosis with PGA sheets. This study is the first to statistically verify the efficacy of PGA sheets for preventing anastomotic leakage in patients with left-sided colon or rectal cancer who underwent DST anastomosis.

Three intraoperative factors associated with anastomotic leakage have been reported: (i) incomplete anastomosis, e.g., thinned rectal wall, inadequate donut, or staple malformation; (ii) tension; and (iii) blood supply [11, 31, 32]. The risk of anastomotic leakage associated with tension and blood supply can be avoided by performing sufficient mobilization for tension-free anastomosis and evaluating blood flow using ICG fluorescence [9–13]. Regarding physical pressure resistance, additional suture reinforcements have been proposed for reliable anastomosis; in other words, a robust anastomosis that is clinically sufficient to prevent leakage [20, 21]. However, the DST anastomosis with the PGA sheet proposed in this study is more effective than the conventional reinforcement methods. The reasons for this are as follows. While the effect of reinforcing sutures at the crossing point of staple lines and the use of a linear stapler with a pre-attached bioabsorbable PGA felt on preventing anastomotic leakage has been reported [20, 21, 33, 34], Ikeda et al. demonstrated that anastomotic leakage is often observed at circular staple lines as well as the crossing points of staple lines [35]. Therefore, sutures at the crossing points of staple lines, or the use of a linear stapler with pre-attached bioabsorbable PGA felt alone are insufficient reinforcements, because circular staple lines cannot be reinforced by these procedures. Based on these facts, it is considered that the DST anastomosis with PGA sheets may be a safer procedure, in that it enables all-round reinforcement, including the crossing points of staple lines. Furthermore, suturing the posterior wall is often technically difficult due to insufficient working space, while suturing in itself is difficult in cases of ultralow anastomosis. In contrast, DST anastomosis with PGA sheets does not require training and can be easily performed by any surgeon, even in cases of lower anastomosis.

In addition to the prevention of anastomotic leakage, no anastomotic bleeding was observed in the PGA sheet group. Although this was not the main purpose of using a PGA sheet, their use may have prevented anastomotic bleeding by stabilizing staple formation and promoting wound healing.

The only disadvantage of DST anastomosis with a PGA sheet is that it interferes with the identification of failure points when the intraoperative leakage test is positive. However, the leakage point could still be identified by flipping the sheet. We experienced one case of positive intraoperative leakage test, but no postoperative anastomotic leakage occurred by identifying the leakage point and reinforcing it with additional intracorporeal sutures.

This study has several limitations. First, this was a retrospective study with a small cohort in a single center. Second, improvements in the circular stapler over the last few years may have contributed to the prevention of anastomotic leakage. Third, although no patients developed anastomotic stenosis, long-term surveillance for anastomotic stenosis was not performed. Fourth, a variety of patient-related factors such as, smoking and anemia may contribute to the development of anastomotic leakage, out of which only ASA-PS and diabetes mellitus were evaluated in this study.

## Conclusion

In conclusion, it has been clarified that DST anastomosis with the PGA sheet, which is easy to perform, contributes to the reduction of anastomotic leakage rate by increasing the strength of anastomosis.

## Data Availability

The datasets used and/or analyzed during the current study are available from the corresponding author upon reasonable request.

## Abbreviations

DST: double-stapling technique
PGA: polyglycolic acid
ICG: indocyanine green
CT: computed tomography
NACRT: neoadjuvant chemoradiotherapy
Ra: tumor above the peritoneal reflection
Rb: tumor at or below the peritoneal reflection
N/A: not applicable

## References

[1] Kuramoto M, Ikeshima S, Yamamoto K, Morita K, Uchihara T, Itouyama R, Yoshimatsu S, Shimada S, Baba H (2017). The intentional oblique transection double stapling technique in anterior resection for rectal cancer. Surg Today.

[2] Nagaoka T, Yamaguchi T, Nagasaki T, Akiyoshi T, Nagayama S, Fukunaga Y, Chino A, Ishizuka N, Konishi T (2021). Safety of small circular Staplers in double stapling technique anastomosis for sigmoid Colon and rectal Cancer. Dis Colon Rectum.

[3] Paun BC, Cassie S, MacLean AR, Dixon E, Buie WD (2010). Postoperative complications following surgery for rectal cancer. Ann Surg.

[4] Kawada K, Hasegawa S, Hida K, Hirai K, Okoshi K, Nomura A, Kawamura J, Nagayama S, Sakai Y (2014). Risk factors for anastomotic leakage after laparoscopic low anterior resection with DST anastomosis. Surg Endosc.

[5] Watanabe T, Miyata H, Konno H, Kawai K, Ishihara S, Sunami E, Hirahara N, Wakabayashi G, Gotoh M, Mori M (2017). Prediction model for complications after low anterior resection based on data from 33,411 japanese patients included in the National Clinical Database. Surgery.

[6] Wang S, Liu J, Wang S, Zhao H, Ge S, Wang W (2017). Adverse Effects of Anastomotic Leakage on local recurrence and Survival after curative anterior resection for rectal Cancer: a systematic review and Meta-analysis. World J Surg.

[7] Koedam TWA, Bootsma BT, Deijen CL, van de Brug T, Kazemier G, Cuesta MA, Fürst A, Lacy AM, Haglind E, Tuynman JB, Daams F, Bonjer HJ, COLOR COLOR II study group (2022). Oncological outcomes after anastomotic leakage after surgery for Colon or rectal Cancer: increased risk of local recurrence. Ann Surg.

[8] Lu ZR, Rajendran N, Lynch AC, Heriot AG, Warrier SK (2016). Anastomotic leaks after restorative resections for rectal Cancer Compromise Cancer Outcomes and Survival. Dis Colon Rectum.

[9] Nowakowski M, Małczak P, Mizera M, Rubinkiewicz M, Lasek A, Wierdak M, Major P, Budzyński A, Pędziwiatr M (2018). The safety of selective use of Splenic Flexure mobilization in sigmoid and rectal resections-systematic review and Meta-analysis. J Clin Med.

[10] Ludwig KA, Kosinski L (2012). Is splenic flexure mobilization necessary in laparoscopic anterior resection? Another view. Dis Colon Rectum.

[11] Watanabe J, Ishibe A, Suwa Y, Suwa H, Ota M, Kunisaki C, Endo I (2020). Indocyanine green fluorescence imaging to reduce the risk of anastomotic leakage in laparoscopic low anterior resection for rectal cancer: a propensity score-matched cohort study. Surg Endosc.

[12] Yanagita T, Hara M, Osaga S, Nakai N, Maeda Y, Shiga K, Hirokawa T, Matsuo Y, Takahashi H, Takiguchi S (2021). Efficacy of intraoperative ICG fluorescence imaging evaluation for preventing anastomotic leakage after left-sided colon or rectal cancer surgery: a propensity score-matched analysis. Surg Endosc.

[13] Peltrini R, Podda M, Castiglioni S, Di Nuzzo MM, D’Ambra M, Lionetti R, Sodo M, Luglio G, Mucilli F, Di Saverio S, Bracale U, Corcione F (2021). Intraoperative use of indocyanine green fluorescence imaging in rectal cancer surgery: the state of the art. World J Gastroenterol.

[14] Matsumoto T, Hamada M, Inada R, Yoshida T, Kobayashi T, Taniguchi N, Oishi M, Shigemitsu K, Sekimoto M (2020). The possibility of a transanal tube as an alternative to diverting stoma in terms of preventing severe postoperative anastomotic leakage after laparoscopic low anterior resection. Int J Colorectal Dis.

[15] Chen H, Cai HK, Tang YH (2018). An updated meta-analysis of transanal drainage tube for prevention of anastomotic leak in anterior resection for rectal cancer. Surg Oncol.

[16] Matsuda M, Tsuruta M, Hasegawa H, Okabayashi K, Kondo T, Shimada T, Yahagi M, Yoshikawa Y, Kitagawa Y (2016). Transanal drainage tube placement to prevent anastomotic leakage following colorectal cancer surgery with double stapling reconstruction. Surg Today.

[17] Herzig DO, Ogilvie JW, Chudzinski A, Ferrara A, Ashraf SQ, Jimenez-Rodriguez RM, Van der Speeten K, Kinross J, Schimmelpenning H, Sagar PM, Cannon JA, Schwiers ML, Singleton DW, Waggoner JR, Fryrear R, Sylla P (2020). Assessment of a circular powered stapler for creation of anastomosis in left-sided colorectal surgery: a prospective cohort study. Int J Surg.

[18] Sylla P, Sagar P, Johnston SS, Dwarakanathan HR, Waggoner JR, Schwiers M, Roy S (2022). Outcomes associated with the use of a new powered circular stapler for left-sided colorectal reconstructions: a propensity score matching-adjusted indirect comparison with manual circular staplers. Surg Endosc.

[19] Pla-Martí V, Martín-Arévalo J, Moro-Valdezate D, García-Botello S, Mora-Oliver I, Gadea-Mateo R, Cozar-Lozano C, Espí-Macías A (2021). Impact of the novel powered circular stapler on risk of anastomotic leakage in colorectal anastomosis: a propensity score-matched study. Tech Coloproctol.

[20] Maeda K, Nagahara H, Shibutani M, Ohtani H, Sakurai K, Toyokawa T, Muguruma K, Tanaka H, Amano R, Kimura K, Sugano K, Ikeya T, Iseki Y, Hirakawa K (2015). Efficacy of intracorporeal reinforcing sutures for anastomotic leakage after laparoscopic surgery for rectal cancer. Surg Endosc.

[21] Jiang TY, Zang L, Dong F, Feng B, Zong YP, Sun J, Liu HS, Zheng MH, Ma JJ (2021). Effect of different reinforcement methods on anastomotic leakage prevention after laparoscopic double anastomosis. J Surg Oncol.

[22] Shibutani M, Nagahara H, Fukuoka T, Iseki Y, Okazaki Y, Hirakawa K, Ohira M (2021). Prevention of anastomotic leakage using a polyglycolic acid sheet in double-stapling technique anastomosis for rectal surgery. Ann Med Surg (Lond).

[23] Kanai E, Matsutani N, Aso T, Yamamoto Y, Sakai T (2020). Long-term effects of pleural defect repair using sheet materials in a canine model. Gen Thorac Cardiovasc Surg.

[24] Takagi T, Tsujimoto H, Torii H, Ozamoto Y, Hagiwara A (2018). New polyglycolic acid fabric for the prevention of postoperative pancreatic fistulas. Asian J Surg.

[25] Kadomatsu Y, Fukui T, Mori S, Chen-Yoshikawa TF, Wakai K (2021). Polyglycolic acid sheet covering to prevent recurrence after surgery for spontaneous pneumothorax: a meta-analysis. Sci Rep.

[26] Saito Y, Omiya H, Shomura Y, Minami K, Imamura H (2002). A new bioabsorbable sleeve for staple-line reinforcement: report of a clinical experience. Surg Today.

[27] Zhang W, Wei Z, Che X (2020). Effect of polyglycolic acid mesh for prevention of pancreatic fistula after pancreatectomy: a systematic review and meta-analysis. Med (Baltim).

[28] Jang JY, Shin YC, Han Y, Park JS, Han HS, Hwang HK, Yoon DS, Kim JK, Yoon YS, Hwang DW, Kang CM, Lee WJ, Heo JS, Kang MJ, Chang YR, Chang J, Jung W, Kim SW (2017). Effect of Polyglycolic Acid Mesh for Prevention of Pancreatic Fistula following distal pancreatectomy: a Randomized Clinical Trial. JAMA Surg.

[29] Kimura M, Terashita Y (2019). Use of bioabsorbable staple reinforcement material in side-to-side anastomoses: suture line reinforcement of the weak point of the anastomosis. Ann Med Surg (Lond).

[30] Kimura M, Kuwabara Y, Mitsui A, Katada T, Nagasaki T, Imagami T, Eguchi Y (2021). Reduction in Anastomotic Leakage using Bioabsorbable Material with a circular stapler in a Porcine Model. Indian J Surg.

[31] Kawada K, Sakai Y (2016). Preoperative, intraoperative and postoperative risk factors for anastomotic leakage after laparoscopic low anterior resection with double stapling technique anastomosis. World J Gastroenterol.

[32] Chekan E, Whelan RL (2014). Surgical stapling device-tissue interactions: what surgeons need to know to improve patient outcomes. Med Devices (Auckl).

[33] Yamamoto S, Kanai T, Osumi K, Yo K, Takano K, Tsutsui M, Nakanishi R, Yoshikawa Y, Kaneko Y, Nakagawa M (2017). Anastomotic leakage using Linear Stapling device with pre-attached Bioabsorbable Polyglycolic Acid Felt after laparoscopic anterior resection. Anticancer Res.

[34] Naito M, Yamanashi T, Nakamura T, Miura H, Tsutsui A, Sato T, Watanabe M (2017). Safety and efficacy of a novel linear staple device with bioabsorbable polyglicolic acid felt in laparoscopic colorectal surgery. Asian J Endosc Surg.

[35] Ikeda T, Kumashiro R, Taketani K, Ando K, Kimura Y, Saeki H, Oki E, Morita M, Akahoshi T, Hashizume M, Maehara Y (2015). Endoscopic evaluation of clinical colorectal anastomotic leakage. J Surg Res.

